# The Social and Clinical Factors Associated With Mental Health Act (MHA) Use Among Children and Adolescent Inpatients: A Cohort Study Using Electronic Health Records

**DOI:** 10.1192/bjo.2024.85

**Published:** 2024-08-01

**Authors:** Susan Walker, Daniela Fonseca Freitas, Johnny Downs, Patrick Nyikavaranda, Sonia Johnson

**Affiliations:** 1 Division of Psychiatry, University College London (UCL), London, United Kingdom; 2 Institute of Psychiatry, Psychology and Neuroscience, King's College London, London, United Kingdom; 3 Department of Psychiatry, University of Oxford, Oxford, United Kingdom; 4 Institute of Psychiatry, Psychology and Neuroscience, King's College London, London, United Kingdom; 5 Department of Primary Care & Public Health, Brighton & Sussex Medical School, University of Sussex, Brighton, United Kingdom

## Abstract

**Aims:**

Little is known about the use of the Mental Health Act (MHA) in children and young people (CYP). There is some evidence that having a diagnosis of psychosis or substance misuse disorder, having an intellectual disability, being older and being of black ethnicity are associated with involuntary admission. However, the existing literature is limited and relies on retrospective case note review or surveys based on a small number of sites over short periods of time. We investigated the social and clinical factors associated with MHA use in CYP using electronic health records. We hypothesised that older adolescence, psychosis, more severe illness, the presence of risk to others and Black ethnicity would be associated with involuntary admission under the MHA.

**Methods:**

Using data from the Clinical Record Interactive Search (CRIS) system for South London and the Maudsley (SLaM) services we identified 2165 CYP under 18 years, with a first admission to inpatient units between 2007 and 2021 with complete data on variables of interest; 1638 (75.7%) were voluntary patients for the duration of the admission and 527 (25.3%) had been detained under a section 2 or 3 of the MHA during the admission. We conducted univariable logistic regression to investigate the association between clinical factors (diagnosis, severity of illness, risk) and social factors (gender, age, ethnicity, deprivation) with the outcome i.e. MHA admission. We then conducted multivariable logistic regression to investigate the association between the clinical and social factors and involuntary admission.

**Results:**

In multivariable analyses we found evidence that a diagnosis of psychosis (OR 2.63, 95% CI 1.83–3.76, p < 0.001), being older (age 13–15 years: OR 5.88, 95% CI 3.46–10.03, p < 0.001; age 16–17 years: OR 6.72, 95% CI 3.97–11.41, p < 0.001), having a developmental disorder (OR 1.60, 95% CI 1.04–2.47, p = 0.033) and being of Black ethnicity (OR 2.14, 95% CI 1.60–2.89, p < 0.001) were associated with involuntary admission after accounting for other factors. Being less impaired (i.e. a higher CGAS score) was associated with a lower odds of involuntary admission (moderate impairment: OR 0.56, 95% CI 0.42–0.74, p < 0.001; lowest impairment: OR 0.41, 95% CI 0.30–0.54, p < 0.001).

**Conclusion:**

In this large cohort of child and adolescent inpatients from South East London, we found that CYP of Black ethnicity are more likely than those from White groups to have an involuntary than voluntary psychiatric hospitalisation, after adjusting for social and clinical factors relating to admission. The finding that Black CYP are more than twice as likely to experience involuntary admission is in keeping with prior literature in CYP and the adult literature. This racial inequity requires further investigation to address disparities in access to mental health care and application of the MHA.

